# Using Human‐Induced Pluripotent Stem Cell Derived Neurons on Microelectrode Arrays to Model Neurological Disease: A Review

**DOI:** 10.1002/advs.202301828

**Published:** 2023-10-20

**Authors:** Shiya Lv, Enhui He, Jinping Luo, Yaoyao Liu, Wei Liang, Shihong Xu, Kui Zhang, Yan Yang, Mixia Wang, Yilin Song, Yirong Wu, Xinxia Cai

**Affiliations:** ^1^ State Key Laboratory of Transducer Technology Aerospace Information Research Institute Chinese Academy of Sciences Beijing 100190 China; ^2^ University of Chinese Academy of Sciences Beijing 100049 China; ^3^ The State Key Lab of Brain‐Machine Intelligence Zhejiang University Hangzhou 321100 China

**Keywords:** CMOS‐MEA, human‐induced pluripotent stem cell‐derived neurons (hiPSC‐DNs), in vitro brain‐machine interface (in BMI), microelectrode arrays (MEAs), neural diseases, neurotoxic substances, organoids

## Abstract

In situ physiological signals of in vitro neural disease models are essential for studying pathogenesis and drug screening. Currently, an increasing number of in vitro neural disease models are established using human‐induced pluripotent stem cell (hiPSC) derived neurons (hiPSC‐DNs) to overcome interspecific gene expression differences. Microelectrode arrays (MEAs) can be readily interfaced with two‐dimensional (2D), and more recently, three‐dimensional (3D) neural stem cell‐derived in vitro models of the human brain to monitor their physiological activity in real time. Therefore, MEAs are emerging and useful tools to model neurological disorders and disease in vitro using human iPSCs. This is enabling a real‐time window into neuronal signaling at the network scale from patient derived. This paper provides a comprehensive review of MEA's role in analyzing neural disease models established by hiPSC‐DNs. It covers the significance of MEA fabrication, surface structure and modification schemes for hiPSC‐DNs culturing and signal detection. Additionally, this review discusses advances in the development and use of MEA technology to study in vitro neural disease models, including epilepsy, autism spectrum developmental disorder (ASD), and others established using hiPSC‐DNs. The paper also highlights the application of MEAs combined with hiPSC‐DNs in detecting in vitro neurotoxic substances. Finally, the future development and outlook of multifunctional and integrated devices for in vitro medical diagnostics and treatment are discussed.

## Graphical abstract

This review emphasizes the role of microelectrode arrays (MEAs) in analyzing neural disease models with human‐induced pluripotent stem cell derived neurons (hiPSC‐DNs). It discusses MEA's importance in culturing and detecting signals in hiPSC‐DNs. Various in vitro neural disease models, such as epilepsy and autism spectrum developmental disorder (ASD), are explored. Additionally, MEA and hiPSC‐DNs' applications in detecting in vitro neurotoxic substances are highlighted. The review also explores the potential of multifunctional devices for in vitro medical diagnostics and treatment.

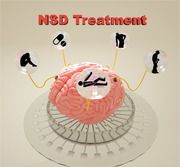

## 1 Introduction

Currently, the establishment of human neural disease models adopt both in vivo and in vitro animal models. However, only about one‐third of animal models provide reliable support or translate to effective human clinical trials, because they cannot accurately recapitulate human physiology.^[^ ^1^, ^2^, ^3^ ^]^ Therefore, there is an urgent need for neural disease models that accurately mimic human physiology. However, ethical and technical constraints limit human experiments and access to human tissue.^[^ ^4^, ^5^ ^]^ Fortunately, the technique of human induced pluripotent stem cells (hiPSCs) has opened up new research possibilities for recapitulating human physiology and microenvironments in vitro,^[^ ^6^ ^]^ particularly through the development of stem cell‐derived organ‐on‐chips.^[^ ^7^ ^]^ In 2006, Japanese scientists Shinya Yamanaka et al. successfully induced mouse skin fibroblasts into iPSCs with the same proliferation and differentiation ability as embryonic stem cells by introducing four factors, Oct3/4, Sox2, c‐Myc, and Klf4, under embryonic stem cell culture conditions.^[^ ^8^ ^]^ This technique revolutionizes the direct reprogramming of mature somatic cells into pluripotent stem cells in vitro, eliminating the requirement for embryos.^[^ ^9^ ^]^ Moreover, hiPSCs can be generated from a patient's own somatic cells, resulting in cells that are genetically matched to the patient, facilitating the research of pathogenesis^[^ ^10^ ^]^ and precise drug screening.^[^ ^11^ ^]^ More recently, the two‐dimensional (2D) neural cultures and three‐dimensional (3D) human brain organoids derived from hiPSC‐derived neurons (hiPSC‐DNs) have been demonstrated as a promising platform for in vitro modeling of the human nervous system.^[^ ^12^ ^]^ Furthermore, the neurological disorders models based on patient‐induced pluripotent stem cell‐derived neurons (piPSC‐DNs) have been progressively refined,^[^ ^13^ ^]^ facilitating the investigation of pathogenic mechanisms,^[^ ^13^, ^14^, ^15^ ^]^ disease prediction,^[^ ^16^ ^]^ and drug screening^[^ ^17^, ^18^, ^19^ ^]^ for neurological disorders in vitro. Moreover, compared to the intact brain, in vitro cultured neurons have a reduced blood–brain barrier, making it more amenable for in situ detection of drug molecules' effects on neural tissue's side effects or efficacy. Additionally, in vitro models enable the investigation of brain metabolism of neuroactive drugs.^[^ ^20^ ^]^ Therefore, the ability to achieve real‐time, in situ detection with high spatiotemporal resolution becomes a crucial metric for evaluating in vitro Brain‐Machine Interfaces (inBMIs). Higher spatial resolutions provide clarity on the connectivity between neural pathways, and lower temporal delays help establish causal relationships in circuit discharge.^[^ ^21^ ^]^ Currently, in vitro neural detection technologies that can achieve high spatial resolution include patch‐clamp, calcium imaging, and microelectrode arrays (MEAs). Patch‐clamp offers high precision and signal‐to‐noise ratio for single neuron detection, whereas MEA surpasses it by simultaneously detecting electrophysiological activities of neural networks at multiple sites in a noninvasive and high‐throughput manner.^[^ ^22^, ^23^, ^24^ ^]^ Similarly, despite achieving precise cellular‐level spatial resolution,^[^ ^25^ ^]^ calcium imaging, even with the fastest GCaMP 8, still exhibits a peak rise time of approximately 20 ms and a half decay of around 150 ms.^[^ ^26^ ^]^ In contrast, MEA provides submillisecond temporal resolution, particularly with the advancements made in CMOS‐MEA technology, which enables subcellular spatial resolution.^[^ ^27^, ^28^, ^29^, ^30^, ^31^ ^]^ Moreover, this technology has been employed to track action potentials propagation across throughout mammalian axonal arbors,^[^ ^32^ ^]^ and resolve propagating neuronal activity in retinal network oscillations.^[^ ^33^ ^]^ More recently, high density CMOS MEAs have been used to map and quantify functional neuronal circuitry in 3D stem cell derived human brain organoids that recapitulate key features observed in vivo brain networks.^[^ ^34^ ^]^ Additionally, when integrated with electrochemistry,^[^ ^35^ ^]^ optics, MEAs enable multidimensional synchronous detection of the electrical activity, neurotransmitter,^[^ ^36^ ^]^ and morphology. As a result, MEA has been widely employed in various in vitro studies of neural diseases, facilitating the detection of neural electrical signals.^[^ ^37^ ^]^

**Figure 1:**
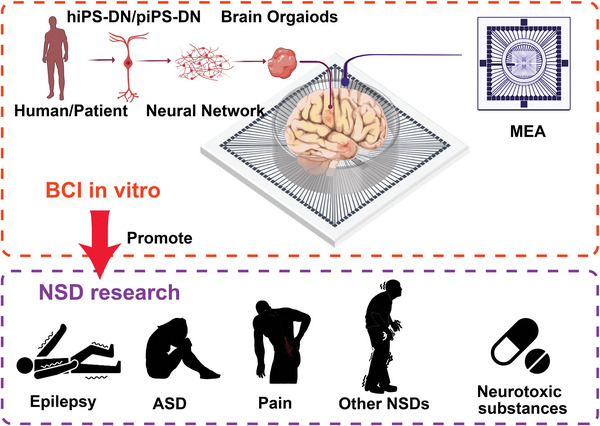
In vitro brain‐machine interfaces (inBMI) in vitro promotes neural disease research. Microelectrode array (MEA) in vitro and human‐induced pluripotent stem cell derived neurons (hiPSC‐DNs) promote neural disease research including epilepsy, ASD, pain, other neural diseases, and neurotoxic substances.

This paper provides a comprehensive review of the literature on the application of hiPSC‐DNs and MEA in neural disease research, consolidating and summarizing the previously scattered work. Firstly, we emphasize the significance of inBMI, particularly the MEA, in neural disease research. We also discuss the cultivation of hiPSC‐DNs and the fabrication of MEA in vitro. Secondly, we focus on the utilization of MEA in neural disease research, encompassing epilepsy, autism spectrum developmental disorder (ASD), and other neural diseases (**Figure** **1**). Furthermore, the hiPSC‐DN model is not only valuable for drug screening, but also for evaluating the efficacy of neurotoxic substances that remain poorly understood. Finally, we explore the future development prospects of MEA, in conjunction with its application in hiPSC‐DNs.^[^ ^38^, ^39^, ^40^, ^41^ ^]^

## 2 Establishment of an In Vitro Human Brain Detection Platform

### 2.1 Culturing of In Vitro Human Brain

**Figure 2:**
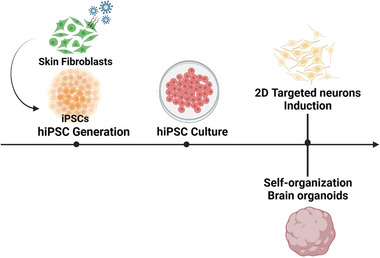
Acquisition of targeted human‐induced pluripotent stem cell derived neurons (hiPSC‐DNs). Human skin fibroblasts were reprogrammed to obtain hiPSCs, and the target hiPSC‐DNs were obtained after hiPSC culture and differentiation.

The generation of in vitro human brain models involves a series of steps, including generation, culture, differentiation, and organization (**Figure** **2**). Above all, hiPSCs can be purchased from commercial hiPSC lines or generated from a wide range of somatic cells, including adult skin fibroblasts, adipocytes, and keratinocytes.^[^ ^42^, ^43^, ^44^ ^]^

HiPSC‐DNs can be differentiated into either 2D neural tissue or 3D brain‐like organoids.^[^ ^45^ ^]^ 2D neural tissue provides a rapid and high‐throughput experimental platform, while 3D brain‐like organoids closely resemble the structure and function of real brain tissue. In terms of cultivation, 3D brain‐like organoids require the utilization of self‐organization properties of neural stem cells on the basis of 2D neural tissue, with specific details and steps elaborated below.

#### 2.1.1 Classification of In Vitro Human Brain Models

One of the methods for obtaining sustainable human 2D neural tissues is to cultivate hiPSC‐DNs on a 2D substrate. They provide a simplified cellular system that allows for more precise detection of electrophysiological or electrochemical changes in neural populations,^[^ ^46^ ^]^ such as conducting in situ drug screening while excluding the blood‐brain barrier and other brain region interventions.^[^ ^47^ ^]^ They also have been utilized for cellular‐level studies in diseases such as Alzheimer's disease^[^ ^48^ ^]^ and Parkinson's disease.^[^ ^49^ ^]^ However, this approach may not be sufficient for the establishment of realistic neural models.

Luckily, 3D brain organoids are self‐organized 3D structures composed of multiple types of hiPSC‐derived cells, aiming to mimic the structure and function of the human brain or specific brain regions.^[^ ^50^ ^]^ They possess cellular hierarchy and tissue microenvironments, enabling the study of coordinated interactions between different cell types.^[^ ^51^ ^]^ They have been used to model diseases such as microcephaly disorders in patients,^[^ ^52^ ^]^ autism caused by SHANK3 deficiency,^[^ ^53^ ^]^ and Miller–Dieker syndrome caused by heterozygous deletion of chromosome 17p13.3.^[^ ^54^ ^]^ These 2D neural tissues and 3D brain organoids provide valuable models for studying the pathogenesis of neurological disorders, drug screening, and personalized medicine,^[^ ^49^ ^]^ offering insights into disease mechanisms and potential therapeutic interventions.

#### 2.1.2 Induction of In Vitro Human Brain

The culturing methods of 2D neural tissue and 3D brain organoids for hiPSC‐DNs in vitro have both overlapping and distinct steps. The shared steps encompass culturing and maintaining hiPSCs, and further differentiating into mature neurons. In contrast to the formation of 2D neural tissues, the generation of 3D organoids requires suspension or gel‐based culture methods.^[^ ^55^ ^]^ Differentiated cells derived from iPSCs can self‐organize into 3D structures on scaffolds such as hydrogels,^[^ ^56^ ^]^ fibrin,^[^ ^57^ ^]^ and carbon fibers.^[^ ^58^ ^]^ By manipulating culture conditions, signaling molecules, and biophysical environments, these scaffolds guide the development of various 3D structures, including neurospheres,^[^ ^59^ ^]^ embryoid bodies,^[^ ^60^ ^]^ forebrain organoids, midbrain organoids, and cortical organoids,^[^ ^61^, ^62^, ^63^ ^]^ which exhibit specific tissue organization and functionality. The specific description of the overlapping steps in both culture methods is as follows.

Above all, the hiPSC‐DNs from skin fibroblasts is used most commonly. Adult skin fibroblasts can be genetically reprogrammed that four transcription factors Oct3/4, Sox2, c‐Myc, and Klf4 are inserted into DNA via retrovirus to obtain hiPSC line.^[^ ^8^ ^]^ In addition, binding plasmids encoding Oct3/4, Sox2, Klf4, L‐Myc, LIN28, and TP53 shRNAs can also efficiently generate hiPSCs.^[^ ^64^ ^]^ The generated hiPSCs can be aligned to a healthy human hiPSC library to ensure successful induction of hiPSCs.^[^ ^65^ ^]^ If the patient‐specific hiPSCs are required, skin fibroblasts can be obtained from the patient to generate piPSCs. However, owing to the differences in the vulnerability of cells extracted from patients with different neural diseases, transcription factors in gene reediting need to be selected according to disease type. For example, inappropriately induced piPSCs with Parkinson's disease may be more vulnerable than hiPSCs in healthy individuals, and may even cause pathological ɑ‐synuclein accumulation. Therefore, selecting reasonable transcription factors including LIN28, shp53, EBNA1, p53DD, and GLIS133 except the common four factors can avoid the vulnerability of piPSCs.^[^ ^66^ ^]^

In addition, the medium usually requires the addition of 0.1 × 10^−3^ m nonessential amino acid, 0.1 × 10^−3^ m β‐mercaptoethanol, 20% knockout serum replacement, 0.1 × 10^−3^ m l‐glutamine 5 ng mL^−1^ basic fibroblast growth factor (bFGF), and recombinant E8 fragments of laminin isoforms which could promote greater adhesion and long‐term self‐renewal of hiPSCs.^[^ ^43^, ^67^ ^]^ Among them, bFGF is an important component of the medium, which keeps the cells in an undifferentiated state in a serum‐free medium to maintain the ability of infinite proliferation. A chemokine CCL2 involved in the immune response was reported to replace bFGF and enhances the totipotency of stem cells.^[^ ^68^ ^]^ After 2–3 days of expansion, the medium on the cells was replaced with induction medium for 6 days in hypoxic (5% O_2_) conditions. The cells were then further expanded in specification medium for 2–3 days.^[^ ^69^ ^]^ These steps are applicable to hiPSC‐derived microglia and brain microvascular endothelial‐like cells as well.

Furthermore, the hiPSCs are differentiated into different target neurons by suitable neural induction medium, including N_2_ medium, nerve‐inducing substances, nutritional factors (e.g., nonessential amino acids, vitamins), and antibiotics (e.g., penicillin, streptomycin). Subsequently, the hiPSC‐DNs were judged whether differentiate into the target neuron, including the morphology of the neuron and the functional protein specifical expression. Finally, the hiPSC‐DNs were isolated and collected to the surface of MEA.

The above‐mentioned common steps apply to the cultivation of hiPSCs, however, the methods for cultivating different types of hiPSC‐derived neurons vary, including dopaminergic,^[^ ^70^ ^]^ glutamatergic,^[^ ^71^ ^]^ cholinergic,^[^ ^72^ ^]^ GABAergic,^[^ ^73^ ^]^ serotonin‐activated neurons^[^ ^74^ ^]^ and neural precursor cells under a combination of growth factors and cell culture conditions^[^ ^75^ ^]^ as well as supporting cells such as astrocytes^[^ ^76^, ^77^ ^]^ and microglia^[^ ^78^ ^]^ in vitro.

### 2.2 In Vitro MEA for hiPSC‐DNs

Thomas et al. invented planar MEA in 1972, laying the foundation for in vitro neural activity detection.^[^ ^79^ ^]^ Groundbreaking worked of Jerome Pine in 1980^[^ ^80^ ^]^ established the basis for the field that led to the establishment of long‐term in vitro recordings and electrical stimulation.^[^ ^81^ ^]^ Subsequently, the first high channel arrays were developed by particle physicists Alan Litke in 2004.^[^ ^82^ ^]^ The combination of these technological breakthroughs have enabled breakthroughs in high‐density recording technology built on the shoulders of the silicon computer chip industry.^[^ ^83^ ^]^ This technology has paved the way to high density CMOS based microelectrode arrays. Moreover, the development of 3D MEA technology has laid the foundation for enabling extracellular stimulation and recording within tissue,^[^ ^84^ ^]^ and more recently, flexible techniques have been integrated with 3D MEA technology to explore the detection of brain organoids.^[^ ^85^ ^]^ Additionally, the combination of microfluidic technology with 3D MEA allows for simultaneous in vitro detection and guided neuronal growth.^[^ ^86^, ^87^ ^]^ Due to the current absence of widely adopted styles and fabrication processes for 3D MEAs, and the fact that planar electrodes are still the most commonly used for in vitro models, research on planar electrodes remains the primary focus at present.

#### 2.2.1 Fabrication of MEA

**Figure 3:**
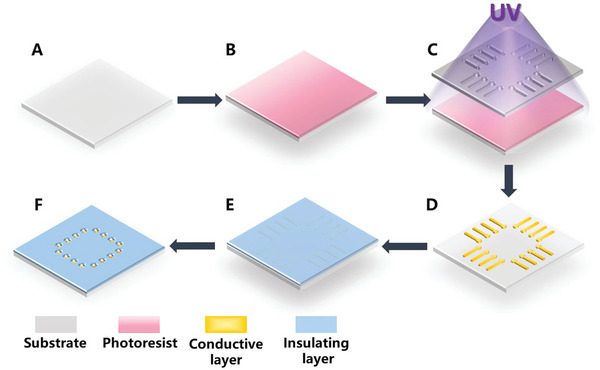
The fabrication of microelectrode arrays (MEA). A) Cleaning the substrate. B) Spin‐coating photoresist. C) The first photolithography to form the conductive layer pattern. D) Sputtering the conductive layer, and the redundant conductive layer was removed by lift‐off process. E) Deposition of an insulating layer. F) The electrodes and pads were etching followed by a second photolithography.

The typical planar MEA fabrication processes employ Micro‐Electro‐Mechanical System (MEMS) technology, and the fabrication steps include cleaning, photolithography, sputtering, stripping, deposition and etching,^[^ ^88^, ^89^, ^90^ ^]^ as shown in **Figure** **3**. Traditional in vitro planar electrodes utilize a glass substrate, while silicon‐based electrodes, commonly used for in vivo applications, can also be employed for implantable detection of 3D organoids.^[^ ^34^ ^]^

Photolithography is a crucial step to obtain the conductive electrode patterns on the MEA.^[^ ^91^, ^92^, ^93^ ^]^ In the first photolithography, the shape of the conductor layer is defined.^[^ ^94^ ^]^ To achieve high‐resolution electrical signal acquisition, high‐density electrode layouts and small electrode spacing are employed. Rational electrode layout and arrangement are crucial to maximize coverage of the target area while minimizing interference between electrodes. In the design, stimulating electrodes are usually larger to efficiently deliver electrical pulses to neural tissue, and they may have either a flat or rounded shape to ensure even stimulation.^[^ ^95^ ^]^ On the contrary, recording electrodes are typically smaller and more delicate to capture subtle electrical signals from individual neurons. Then, the chip is placed inside a vacuum chamber, where metal patterns are deposited onto its surface using methods such as sputtering, e‐beam deposition, or thermal evaporation. And the excess conductive layer is removed by a lift‐off process.^[^ ^96^ ^]^

The insulation can form complete insulating isolation between the conductive layer as solution to avoid signal crosstalk.^[^ ^97^, ^98^, ^99^ ^]^ Typically, the insulating layer can be deposited as SiO_2_/Si_3_N_4_ using the plasma enhanced chemical vapor deposition method. Additionally, Parylene‐C can be applied through a vacuum deposition process, while SU8 can be coated using a spin coating process.^[^ ^100^, ^101^, ^102^ ^]^ In the second photolithography, microelectrodes, reference electrodes and bonding pads are exposed. Oxygen plasma cleaning is commonly performed after completing photolithography to remove fabrication residue impurities on the surface of the chip. Then, the windows on the electrodes and pads are opened to form the electrical connection with neurons, and the process is similar to photolithography. In this step, the etching is carried out to remove the insulating layer.^[^ ^103^, ^104^, ^105^ ^]^

Commercial MEAs have enabled researchers in the neuroscience community to study neuronal signaling at millisecond timescales across hundreds to thousands of sites from in vitro and ex vivo neuronal preparations, as well as stem cell derived models of the central nervous system. Current commercial MEA types include multichannel, porous, and complementary metal oxide semiconductor (CMOS).^[^ ^106^ ^]^ Major companies include MaxWell Biosystems, Axion Biosystems, 3Brain, Multi Channel Systems MCS GmbH, and Med64 (former AlphaMED).

Commercial MEAs currently face challenges in enhancing the biocompatibility of 2D surface materials, and the limited availability of 3D MEA configurations restricts optimal hiPSC interfacing. These technical constraints not only impact the long‐term cell viability on rigid and inorganic MEA surfaces but also hinder the effective recording of signals from 3D organoid surfaces. Therefore, it is urgent to explore the new MEA and apply it to establish the hiPSC‐DN models. This review systematize the hiPSC‐DN model detection, which can improve the BMI in vitro technology.

#### 2.2.2 Structure and Modification of MEA

**Figure 4:**
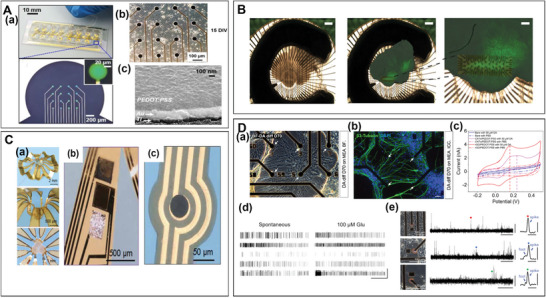
Development of advanced microelectrode arrays (MEAs). A) Compact 256‐channel 16‐well microelectrode.^[^ ^109^ ^]^ a) 256‐channel and 16‐well MEA on a microscope glass slide with PDMS chamber and membrane integration. PEDOT:PSS electrodeposited 16 microelectrode arrays in a unit well. b) Micrographs of hippocampal neurons at 15 DIV. c) Magnified SEM image of PEDOT:PSS deposited electrodes. B) MEA contains 60 platinum 3D‐electrodes.^[^ ^114^ ^]^ a) Left panel: Image of a typical coronal midbrain slice, mounted on the 3D MEA biochip (scale bar: 1 mm). Middle panel: Overlay of the image before recording with the respective image of the same slice, fixed and stained for tyrosine hydroxylase. Right panels: MEA electrodes (scale bars: 250 µm). C) 3D flexible neural interfaces.^[^ ^85^ ^]^ a) Optical micrographs of a 3D flexible neural interface; b) electrochemical oxygen sensor; c) circular microelectrode. D) Dual‐mode MEA.^[^ ^35^ ^]^ a) Bright‐field images of mature dopaminergic neurons cultured on MEA; b) immunocytochemistry images of mature dopaminergic neurons cultured on the surface of MEA (scale bars: 50 µm). c) CV curves of the bare, CNTs/PEDOT:PSS‐modified and rGO/PEDOT:PSS‐modified MEAs. d) Electrophysiological spikes (scale bars: 140 µV, 50 s). e) Amperometric traces of K+‐induced exocytosis spikes were collected by the microelectrodes over different sites. Scale bars of amperometric traces (left): 5 pA, 2 s; Scalebars of spikes (right): 5 pA, 25 ms. A) Reproduced with permission.^[^ ^109^ ^]^ Copyright 2020, Royal Society of Chemistry. B) Reproduced under terms of the CC‐BY license.^[^ ^114^ ^]^ Copyright 2021,The Authors. Published by Frontiers Media S.A. C) Reproduced under terms of the CC‐BY license.^[^ ^85^ ^]^ Copyright 2021, The Authors. Published by AAAS. D) Reproduced with permission.^[^ ^35^ ^]^ Copyright 2022, Elsevier.

To enhance the throughput and quality of the signal detection, MEA has undergone improvements in the number and the surface structure of its electrodes. In terms of throughput enhancement, the number of electrode sites increased from 64 to 128 and 256 (**Figure** **4**B),^[^ ^88^, ^89^, ^107^ ^]^ while the number of wells has also expanded to 1, 6, 9, 24, 48, and 96 wells (Figure 4Aa,b). This enables the simultaneous detection of hiPSC‐DNs' response to different drug matrices.^[^ ^108^, ^109^ ^]^ Subsequently, CMOS‐MEA has significantly boosted the throughput of MEA to 4096 channels, thus overcoming the limitations of traditional planar MEA in temporal and spatial resolution.^[^ ^28^ ^]^

In 2014, the Hierlemann lab further increased the electrode count to 26,400, achieving subcellular spatial resolution.^[^ ^29^ ^]^ Meanwhile, while maintaining a high channel count, the Andreas team continued to develop a CMOS MEA with both high signal‐to‐noise ratio and high throughput, enabling the long‐term detection of iPSC‐derived neuronal cultures for up to several weeks.^[^ ^31^ ^]^ By 2023, the electrode count had reached 236 880, ensuring precise coverage across the cortical and hippocampal brain regions.^[^ ^30^ ^]^ Additionally, high‐density CMOS shanks in Neuropixels probes have recently been employed for signal detection in human brain organoids, offering a method for vertical signal detection in organoids.^[^ ^34^ ^]^

In addition, 3D flexible electrodes have opened up new possibilities for recording across the surface and throughout the volume of brain organoids (Figure 4C).^[^ ^85^, ^100^ ^]^ The vertical structure of MEAs facilitates signal detection from multiple orientations, while surface modifications of electrode sites contribute to enhanced detection capabilities.^[^ ^101^ ^]^ Nanomaterials have been proven effective in improving the electron transfer efficiency of bare metal electrodes, reducing impedance, and increasing the signal‐to‐noise ratio of electrical signal detection. Among these modification materials, metal nanoparticles are the most commonly used and have shown promise in increasing MEA detection sensitivity. In recent years, nanocomposite membranes composed of carbon nanomaterials and conductive polymers have gained popularity for MEA modification due to their superior biocompatibility,^[^ ^110^, ^111^, ^112^, ^113^ ^]^ stability and conductivity. For example, rGO/PEDOT:PSS has been shown to significantly enhance electrode compatibility with hiPSC‐DNs, reduce impedance, and minimize phase delay (Figure 4Ac, Da,b).^[^ ^114^ ^]^

In terms of functionality, aside from the electrophysiological detection, electrochemical detection has also been seamlessly integrated into the MEA (Figure 4D).^[^ ^115^, ^116^, ^117^ ^]^ For example, the use of nafion and oxidase on the modified electrode surface can convert chemical signal induced by neurotransmitter into current signal recorded by MEA. Furthermore, MEAs can be employed for the electrically stimulate of hiPSC‐DNs in vitro. For example, Shahaf and Maron utilized MEAs to investigate the relationship between selective learning and memory of in vitro neuronal networks through electrical stimulation.^[^ ^118^, ^119^ ^]^ Similarly, Steve Potter's group conducted similar work in 2001, where they electrically stimulated neurons in vitro to control virtual environments,^[^ ^120^ ^]^ using electrical stimulation to replace natural neural inputs^[^ ^121^ ^]^ for training the stimulated neurons.^[^ ^122^ ^]^ In addition to using stimulation to facilitate learning, Lana et al. achieved stimulus‐free learning and applied it in a simulated experiment of neuronal wall avoidance in vitro.^[^ ^123^ ^]^ Recently, Kagan et al. have advanced similar work by applying high‐density MEAs for the detection of hiPSCs, demonstrating their learning and self‐organizing capabilities.^[^ ^124^ ^]^ This suggests the possibility of constructing an in vitro neuronal network which can response to an external input from the CMOS chip.

The development of MEA technology and the generation of brain organoids using hiPSC‐DNs will play a pivotal role in advancing this synthetic biological intelligence.^[^ ^125^ ^]^ Additionally, MEA can be effectively combined with other technologies. For instance, immunocytochemistry and high‐content imaging technology have been employed to observe the morphology and growth of hiPSC‐DNs. The combination of these technologies will enable multidimensional detection of hiPSCs, encompassing electrochemical, electrophysiological, morphological.

#### 2.2.3 Physiological Activity of Detection by MEA

The discharge waveforms in the recorded electrophysiological signals obtained from MEA can be used to differentiate neuron subtypes. For example, Buzsaki's lab has developed software that enables the identification and classification of neuron subtypes based on extracellular recordings.^[^ ^126^ ^]^ Additionally, another classification method called WaveMAP, which combines nonlinear dimensionality reduction and graph clustering, can also be employed for neuron classification.^[^ ^127^ ^]^ Other metrics such as inter‐spike intervals (ISIs) distributions, burst to bust similarity cores, and functional connectivity, as well as spatiotemporal dynamics were extensively explored by Sharf et al. in brain organoids.^[^ ^34^ ^]^

The electrophysiological parameters^[^ ^128^ ^]^ which focused on the relationships between neurons and neural networks detected by MEA are shown in **Table** **1**. Among these parameters, one of the fundamental parameters is the mean firing rate (MFR), which can reflect the excitability of neurons. A higher MFR corresponds to increased neuronal excitability.^[^ ^129^ ^]^ At the same time, the shape of the spike waveforms contributes to reveal the type of the detected neurons including excitatory and inhibitory neurons. For the analysis of the neural network, burst‐related metrics such as mean burst rate (MBR) and the number of bursts (NB) can indicate synchronization of neural network. For example, when closely situated MBRs occur, it may indicate the occurrence of synchronized burst activities within the network, which can guide further analysis of the correlation in their discharge times.^[^ ^130^ ^]^ The ISI and inter‐burst interval (IBI) can be used as indicators to assess the occurrence of bursts and network bursts. The ISI and IBI methods are robust for detecting changes in burst or network burst patterns and characteristics induced by environmental alterations.^[^ ^131^ ^]^ The comprehensive use of various electrophysiological parameter lays a foundation for the analysis and establishment of the hiPSC‐DN models.^[^ ^132^ ^]^ Above all, the discharge waveforms in the recorded electrophysiological signals obtained from MEA can be used to differentiate neuron subtypes. Recently, Buzsaki's lab has developed software that enables the identification and classification of neuron subtypes based on extracellular recordings.^[^ ^126^ ^]^ Additionally, another classification method called WaveMAP, which combines nonlinear dimensionality reduction and graph clustering, can also be employed for neuron classification.^[^ ^127^ ^]^ Other metrics such as ISIs distributions, burst to bust similarity cores, and functional connectivity, as well as spatiotemporal dynamics were extensively explored by Sharf et al. in brain organoids.^[^ ^34^ ^]^

**Table 1:** The electrophysiological parameters are commonly used in microelectrode arrays (MEAs) in vitro.

Parameters	Description
Mean firing rate (MFR)	Mean number of spikes normalized by the time of the recording
Weighted mean firing rate (wMFR)	Weight mean number of spikes normalized by the time of the recording
Mean burst rate (MBR)	Mean number of bursts normalized by the time of the recording
Number of bursts (NB)	Total number of single‐electrode bursts throughout the analysis
Number of spikes in a burst	The calculated number of spikes that occur within bursts
Percent of isolated spikes	Percent of spikes occurring outside of bursts
Mean network burst rate (MNBR)	Mean number of bursts normalized in networks by the time of the recording
Number of network bursts	Total number of network bursts throughout the analysis
Number of spikes per network burst	The average number of spikes in a network burst
Burst duration (BD)	Length of time that a burst lasts between the first and last spike
Network burst duration (NBD)	Average time from the first spike to the last spike in a network burst
Inter‐burst interval (IBI)	The time between the trailing spike of each burst and the leading spike of the subsequent burst
IBI coefficient of variation	Variability of the inter‐burst interval. This metric provides a measure of burst rhythmicity bursts occurring at regular intervals have a small coefficient of variation, whereas sporadic bursting has a larger coefficient of variation
Network IBI coefficient of variation	The coefficient of variation (standard deviation/average) for the inter‐network burst interval, the time between network bursts. This is a measure of network burst regularity
Inter spike interval (ISI)	The time interval between the start and end of a spike
ISI coefficient of variation	The coefficient of variation of the inter‐spike intervals for all spikes in the recording. This is a measure of spike regularity
Coefficient of variation (CV) of the ISIs	The difference in time between adjacent spikes in each channel was computed to obtain the ISIs. The mean and standard deviation of the ISIs for each channel was computed to yield a CV
Normalized MAD burst spike number	A calculation of the distance between each data value and the mean to assess the variation in a data set. In this case, it is the statistical dispersion of the spikes within bursts.
Median ISI/Mean ISI	Median ISI divided by the mean ISI

In addition to the neuronal electrophysiological signal detection, neurotransmitter from synapses also is conducive to analyzing the state of neurons.^[^ ^36^, ^117^, ^133^ ^]^ Typically, the waveform and intensity of the current pulses generated by vesicle exocytosis can be recorded by MEA to analyze the quantity of neurotransmitters in the vesicles.^[^ ^35^ ^]^ Therefore, the exocytosis of neurotransmitter vesicles are quantitatively analyzed and the physiological characteristics of hiPSC‐DNs are further revealed.

## 3 In Situ Analysis of Neural Disease Model of hiPSC‐DNs on MEA

The cause of neural diseases is uncertain and varies among patients even with the same symptoms. Therefore, it is very important to study the pathogenesis of patients with specific neurological disorders and find corresponding treatment methods.^[^ ^45^, ^134^ ^]^ The development of neurological disorders is influenced by the interplay between genetic mutations and epigenetic factors.^[^ ^135^ ^]^ Genetic mutations include single‐gene or multigene variations.^[^ ^136^ ^]^ For example, iPSC‐derived dopamine neurons carrying the GBA^N370S^ mutation, a single‐gene mutation associated with Parkinson's disease, exhibit sustained mitochondrial calcium dysregulation and impairments in mature neuronal electrophysiological activity.^[^ ^137^ ^]^ Additionally, iPSC‐derived DA neurons derived from individuals with GBA^N370S^ and LRRK2^G2019S^ mutations release increased levels of dopamine.^[^ ^138^ ^]^ In the case of ALS, iPSC‐derived motor neurons carrying SOD1 mutations associated with the disease show reduced amplitude of delayed rectifier potassium currents.^[^ ^139^ ^]^ Multigene variations are also implicated in neurological disorders such as autism spectrum disorders, involving mutations in genes like SHANK3, NLGN3, NLGN4X, among others. Regarding epigenetic‐driven models of neurological disorders, iPSCs derived from individuals with Alzheimer's disease have been used to study pathological features associated with changes in DNA methylation.^[^ ^140^ ^]^ These models have shed light on the pathogenic mechanisms related to epigenetic regulation, including excessive phosphorylation of TAU protein, elevated levels of β‐amyloid, and activation of GSK3B.^[^ ^141^ ^]^

The inBMI mentioned above is well suited for the study of targeted neural disease research. Thus, we comprehensively summarize the application of inBMI in neural disease research. According to the type of neural disease, we elaborated on the contribution of inBMI in epilepsy, ASD, pain, Parkinson, amyotrophic lateral sclerosis (ALS), etc. In addition, neuroexcitatory or inhibitory drugs have the potential to treat neural disease, therefore we also included neurogenic compounds in Part 3.1.3.^[^ ^142^, ^143^, ^144^, ^145^ ^]^ It is beneficial for researchers to target the next hot spot for inBMI for neural disease research.

### 3.1 Epileptiform Electrical Activity of hiPSC‐DNs Recorded by MEA

#### 3.1.1 Modeling of In Vitro Epilepsy on MEA

The establishment of in vitro epilepsy models can be induced by drugs such as picrotoxin, isoniazid, chlorpromazine, strychnine, gabazine, enoxacin.^[^ ^144^, ^146^, ^147^, ^148^, ^149^ ^]^ The epileptiform electrical activity can be used to judge whether the hiPSC‐DNs are overactive or hyper‐synchronized as a criterion for successfully establishing a in vitro epilepsy model.^[^ ^150^ ^]^ For example, Tukker et al. utilized a total of 768 channels in an MEA to establish an epilepsy model by co‐culturing hiPSC‐DNs and astrocytes with chlorpromazine and enoxacin. In this epilepsy model, MFR, MBR, and mean network bust rate (MNBR) increased significantly with the drug accumulating in a certain range, and principal component analysis also was used to confirmed the sensitivity of hiPSC‐DNs to chlorpromazine, and enoxacin.^[^ ^147^ ^]^

**Figure 5:**
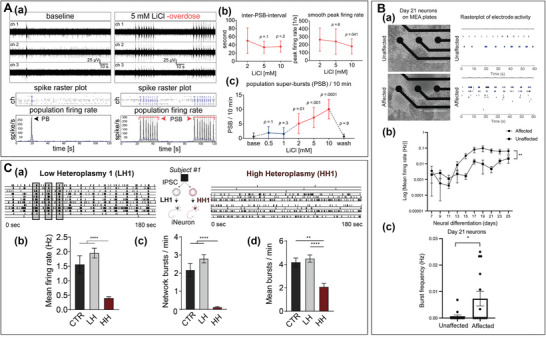
Microelectrode array (MEA) recording results in epilepsy models. A) Overdose concentrations of lithium on human neurons and network function recorded by MEA.^[^ ^151^ ^]^ a) Representative examples of MEA recordings, spike raster plots, and population firing rate diagrams in baseline and overdose concentrations of LiCl. The black arrows are marking the population bursts (PB) and the red arrows with the red line mark the population super‐bursts (PSB). b,c) The properties of PSB activity in overdose concentrations of LiCl. B) An epilepsy‐associated ACTL6B variant captures neuronal hyperexcitability in a human‐induced pluripotent stem cell (hiPSC) model.^[^ ^156^ ^]^ a) Representative images (left) of one well of unaffected and affected neurons differentiated on an MEA. Scale bar, 400 µm. Representative raster plot (right) of network spiking activity after 21 days of maturation. Individual spikes (black); Bursts (blue). b) Time course analysis of the spontaneous neural activity of unaffected and affected neurons of neural differentiation. c) Analysis of burst frequency (Hz) of unaffected and affected neurons on d21 of neural differentiation. C) m.3243A > G‐induced mitochondrial dysfunction impairs hiPSC‐DNs recorded by MEA.^[^ ^155^ ^]^ a) Example plots of LH1,2,3 (normal ‐low heteroplasmy) and HH1,2,3 (impaired‐high heteroplasmy) networks derived from patient 1. b) Mean firing rate (MFR) and network bust rate (NBR) are recorded by MEA. A) Reproduced with permission.^[^ ^151^ ^]^ Copyright 2021, Nature Portfolio. B) Reproduced with permission.^[^ ^156^ ^]^ Copyright 2021, Society for Neuroscience. C) Reproduced with permission.^[^ ^155^ ^]^ Copyright 2020, Elsevier.

Overdose of lithium, antipsychotics chlorpromazine, antibacterial norfloxacin, and isoniazid can trigger seizures, which can also be validated in a inBMI‐established epilepsy model.^[^ ^151^, ^152^, ^153^ ^]^ Izsak et al. studied the effect of lithium on hiPSC‐derived cortical neurons. The addition of lithium salts to the culture medium of hiPSC‐derived cortical neurons resulted in a decrease in ISI and MFR (**Figure** **5**Ab). Simultaneously, MEA recordings exhibited a noteworthy increase in population burst, population super‐burst, and population super‐burst duration (Figure 5Ac). These results are similar to the epileptiform electrical activity in epilepsy models induced by chlorpromazine, enoxacin in vitro. It suggests that excess lithium salts have similar chemically induced epileptiform activity in neural networks. Meanwhile, a specific epileptogenic effect of excess lithium was demonstrated (Figure 5Aa).^[^ ^151^ ^]^

Furthermore, the dynamic stability of activity levels in a neural circuit, specifically the ratio of excitatory to inhibitory neurons in hiPSC‐DNs, will impact the normal functioning and information processing of the neural network. Dysregulation of the ratio of excitatory to inhibitory neurons can lead to neurological abnormalities, such as excessive excitation associated with epileptic seizures or excessive inhibition associated with conditions like depression and anxiety disorders.^[^ ^154^ ^]^ Therefore, altering the ratio of excitatory to inhibitory neurons in hiPSC‐DNs in vitro will directly influence the disease models being constructed.

In conclusion, in vitro models can recapitulate key neurophysiological signatures found epileptic brain tissue in vivo, which can respond to drug‐mediated epileptiform electrical activity. This points to a new milepost for in vitro research and treatment of epilepsy.

#### 3.1.2 Evaluation of Epilepsy Model from piPSC‐DNs on MEA

The iPSC‐DNs stemmed from epilepsy patients can be used to establish specific epilepsy models on MEA in vitro.^[^ ^155^, ^156^, ^157^ ^]^ Patient‐specific models can simulate the in vitro electrophysiological activity of neurons after epilepsy related gene modifications. They also facilitate in vitro drug screening for individual patients. For example, Ahn et al. differentiated piPSC with ACTL6B mutations into neurons and neural progenitor cells to establish EIEE‐76 models (Figure 5Ba). Then a significantly increased MFR and MBR were recorded by MEA in EIEE‐76, indicating that the neuron firing is more active (Figure 5Bb,c). Next, the gene and protein structural variations evaluated by qt‐PCR and Western Blot technology. This multidimensional approach suggests that the gene mutation may contribute to epileptic‐like neuronal discharges. This conclusion was consistent with the clinical finding that biallelic pathogenic mutations in ACTL6B cause early infantile epileptic encephalopathy.^[^ ^156^ ^]^ Also, because insufficient mitochondrial energy is associated with abnormal neural circuit activity.^[^ ^155^, ^158^, ^159^, ^160^ ^]^ Klein et al. differentiated piPSC with mitochondrial encephalopathy (m.3243A >G variant) into cortical neurons to establish models with impaired mitochondrial function.^[^ ^155^ ^]^ In these models, strong decreases in MFRs and NRBs were recorded (Figure 5Cb‐d), which suggest decreased synchrony of neural networks (Figure 5C).^[^ ^155^ ^]^ This work suggests there may be a mechanism linking mitochondrial dysfunction to epilepsy.^[^ ^155^ ^]^ And it also demonstrates that the m.3243A > G mutated neurons produce abnormal firing patterns and neural network function. Similarly, Ichis et al. analyzed the activities of piPSC‐derived GABA neurons with presynaptic syntaxin‐binding protein 1 STXBP1‐E mutation. A significant decrease in neural activity was recorded in STXBP1‐E patient‐derived GABA neurons, including the number of spikes, MFR, and the NB. Combined with the animal experiments, it is concluded that STXBP1‐E mutation is a key factor in GABA neuron dysfunction, further improving the relationship between abnormal expression of this gene and epilepsy.^[^ ^157^ ^]^

Taken together, hiPSC‐DNs have been able to successfully establish epilepsy models in vitro and utilize MEAs to record the electrophysiological signals. Furthermore, the successful epilepsy hiPSC‐DN models have been used for drug assessment, however, if these models are to be further applied in the development of new drugs, the effectiveness and success rate of these models in response to drugs still need to be further verified.

#### 3.1.3 Assessment of Epileptic Effects of Drug on MEA

Drug assessment is to evaluate the toxicity and safety to humans in the later stage of drug development, as a standard to judge whether a drug will induce side effect. The epilepsy models established by hiPSC‐DNs and MEAs in vitro are considered to have the potential for drug assessment. The compounds that have been evaluated in hiPSC‐DNs include picrotoxin(PTX), isoniazid, chlorpromazine, strychnine, gabazine, etc.^[^ ^142^, ^143^, ^144^, ^145^ ^]^ For example, when the PTX in the epilepsy model reached 0.01 × 10^−6^ m, the MBR of hiPSC‐DNs increased significantly and the MNBR increased slightly. Conversely, when PTX concentrations reached 1 or 10 × 10^−6^ m, the MBR was significantly increased, NBR was slightly increased and even epileptic discharge. These results revealed that PTX had epileptogenic effects.^[^ ^143^ ^]^ Therefore, drug concentrations and doses are crucial, and inappropriate dose may lead to epileptiform electrical activity in neurons, which is not expected, resulting in the withdrawal of some drugs from the drug market or the termination of development.^[^ ^142^ ^]^

In addition, the degree of epileptogenicity of drugs in epilepsy models may depend on the proportion of glutamatergic and GABA neurons in hiPSC‐DN models. The ratio of glutamatergic and GABA neurons varying, the MFR of the same epileptic model treated with the same epileptic compound were different.^[^ ^152^ ^]^ Besides the ratio of excitatory to inhibitory neurons,^[^ ^154^ ^]^ factors such as neuronal functional circuit connectivity,^[^ ^34^ ^]^ neural network topology,^[^ ^161^ ^]^ network motif distribution,^[^ ^162^ ^]^ and synaptic connections^[^ ^163^ ^]^ also influence the excitatory/inhibitory balance in the biological brain. Therefore, further enhancing the construction of in vitro human brain models and more comprehensively simulating the excitatory/inhibitory balance mechanisms observed in the biological brain are crucial for drug research.

### 3.2 Synchronized Firing of hiPSC‐DNs Autism Model Recorded by MEA

Autism spectrum disorders (ASDs) are one broad family in a large class of neurodevelopmental disorders. Similar to neural network activities in epilepsy patients, hyperexcitability is a key feature of ASDs.^[^ ^164^, ^165^, ^166^ ^]^ Therefore, the electrophysiology activities of the ASD models can be recorded by inBMI to analyze the pathological process of neurodevelopment disease. Presently, the two risks may cause ASD, including endogenous gene mutation and exogenous compound regulation.^[^ ^167^ ^]^ Hence, the experiment in ASD models focuses on the two directions.

#### 3.2.1 Estimating Neural Electrical Activities of hiPSC with Autism Gene Mutation on MEA

**Figure 6:**
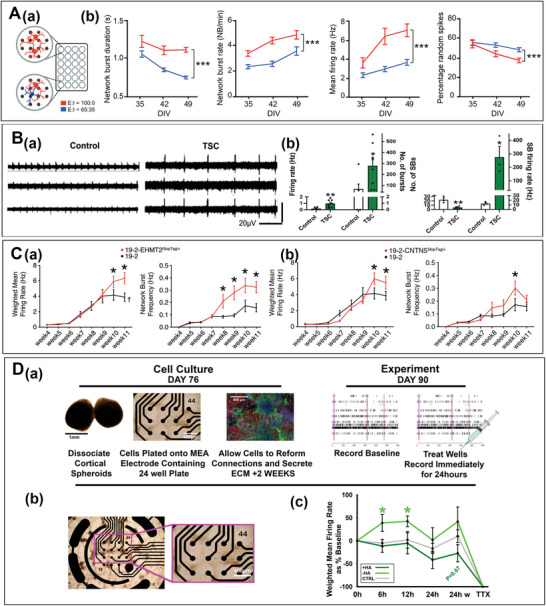
Microelectrode array (MEA) recording of autism spectrum developmental disorder (ASD) models. A) Cadherin‐13 disorder of human‐induced pluripotent stem cells (hiPSCs) derived neuronal networks was recorded by MEA.^[^ ^168^ ^]^ a) iGLUNgn2 alone (E/I ratio: 100:0) or in coculture with iGABAA‐FSK (E: I ratio: 65:35) were recorded by a multiwell MEA. b) Network burst duration; Network burst rate; Mean firing rate; Percentage of random spikes. B) ASD patient‐induced pluripotent stem cell (piPSC) with TSC2 mutation recorded by MEA.^[^ ^169^ ^]^ a) Representative voltage traces from three electrodes of the same MEA culture for control and TSC2 neurons. c) Basal excitability of control and TSC2 neurons showing average spike firing rate and several single unit bursts detected. d) Changes in the synchronized burst (SB) activity and the number of spikes in individual SBs. C) CNTN5‐/+or EHMT2‐/+hiPSC‐DNs (derived neurons) recorded by MEA.^[^ ^170^ ^]^ a) Both weighted mean firing rate (wMFR) and network burst frequency were recorded from the 19–2‐CNTN5 isogenic pair from weeks 4–11 PNI. b) MEA recordings of the isogenic pair 19–2 and 19–2‐EHMT2StopTag/+ iPSC‐DNs from weeks 4–11 PNI. D) Hyaluronic acid (HA) on the regulation of hiPSCs.^[^ ^166^ ^]^ a) Workflow for MEA experiments. b) Brightfield image of dissociated spheroids plated for 2 weeks on a microelectrode array. The inset shows the association of neurons with electrodes. Scale bar 400 µm. c) Quantification of the wMFR. A) Reproduced under terms of the CC‐BY license.^[^ ^168^ ^]^ Copyright 2022, The Authors. Published by Springer Nature. B) Reproduced under terms of the CC‐BY license.^[^ ^169^ ^]^ Copyright 2020, The Authors. Published by Springer Nature. C) Reproduced under terms of the CC‐BY license.^[^ ^170^ ^]^ Copyright 2019, The Authors. Published by eLife Sciences Publications. D) Reproduced under terms of the CC‐BY license.^[^ ^166^ ^]^ Copyright 2020, The Authors. Published by Springer Nature.

Because ASD‐related genes are usually involved in the regulation of the development of neurons and synapses, the mutation of these genes can cause the disorder in brain regions that regulate higher cognitive function. Presently, mutations of Cadherin‐13, ACTL6B, CNTN5, EHMT2, and TSC2 have been confirmed to cause ASD in hiPSC‐DN models. For example, Mossink et al. established an ASD model which contained hiPSC‐derived GABAergic, glutamatergic neurons to elucidate E/I dysregulation in ASD disease caused by the Cadherin‐13 mutation.^[^ ^168^ ^]^ In this ASD model, the high expression of the Cadherin‐13 resulted in an increased proportion of GABA neurons, ultimately presenting the percentage of random spikes (PRS) increase and MFR and MNBR significantly reduced (**Figure** **6**A).^[^ ^168^ ^]^ Additionally, in order to verify the relationship between TSC2 mutation and ASD, Mouhamed et al. established the TSC2 hiPSC‐DNs model on MEA. Then an increase in MFR, the number of spikes in a burst and a significant reduction in MBR were recorded. It indicated that the network activities of the model showed increased excitability but decreased synchrony. Therefore, the TSC2 mutation could imbalance the E/I through the increase in inhibitory synaptic GABA signaling (Figure 6B).^[^ ^169^ ^]^ Not only single‐gene mutation models, but also combinatorial models of gene mutations can be established with hiPSC‐DNs. Eric et al. cultivated a glutamatergic neuron line which is a combinatorial model of the gene (GLI3/KIF21A or EHMT2/UBE2I) mutation.^[^ ^170^ ^]^ The hyperactivities of neural networks in neurons of CNTN5 or EHMT2 deficiency were recorded in these models. And the hyperactivities were manifested by a significant increase in weighted mean firing rate (wMFR) and MNBR. Therefore, inactivation of at least one allele of CNTN5 or EHMT2 can significantly enhanced excitatory neuronal synaptic activity (Figure 6C).^[^ ^170^ ^]^

Furthermore, the application of brain organoids to establish ASD models is an area of ongoing research. Shcheglovitov's lab at Utah has employed brain organoid models of ASD, which simulate neurons with a half‐dose deletion of the SHANK3 gene linked to autism and intellectual disabilities.^[^ ^53^ ^]^ Using MEA, the study confirmed the presence of excitatory and inhibitory synapses and recorded oscillations in the low‐frequency range (4–30 Hz) and mid‐to‐high‐frequency range (30–100 Hz). Moreover, a brain organoid model resembling Rett syndrome, which shares symptomatic similarities with ASD, was established in 2021.^[^ ^171^ ^]^ These organoids exhibited highly abnormal and epileptic‐like activity, recorded by MEA, and were utilized as a tool to detect the effects of the neuroregulatory drug pifithrin‐α on organoid physiological activity.

#### 3.2.2 Drug Regulation Analysis of hiPSC Autism Model on MEA

Because the severity of ASD is associated with molecular alterations in excitatory cortical neurons, cortico‐cortical projection neurons and microglia, it is worth to investigate how ASD is regulated by exogenous compounds at a network level. For example, a 3D cortical spheroid ASD model has been established to explore the effect of hyaluronan on neural marker expression. A decrease in wMFR when hyaluronic acid (HA) was added and a significantly increase in wMFR and an enhancement of the network activity when HA was removed were recorded by MEA. It suggested that HA can prevent hyperexcitability during neural network development. Meanwhile, combined with mRNA nanostring analysis, it was shown that the reduction of HA can increase the expression of excitatory synaptic markers, increase the excitatory synapses, and reduce the formation of inhibitory synapses, thereby balancing excitatory and inhibitory signals (Figure 6D).^[^ ^166^ ^]^ In addition, a compound, the proinflammatory cytokine IL^[^ ^172^ ^]^ is thought to be associated with the severity of ASD^[^ ^173^ ^]^ and has been found in ASD patient plasma,^[^ ^174^, ^175^, ^176^ ^]^ postmortem brain,^[^ ^174^, ^175^, ^176^ ^]^ cerebrospinal fluid^[^ ^177^ ^]^ and ASD mouse models.^[^ ^178^ ^]^ Accordingly, Baldino Russo et al. establish the model to explore the effect of IL‐6 on the development of neuronal synapses, which consisted of co‐culture of iPSC‐derived neurons and glial cells derived from ASD patients. In this ASD model, when blocking IL‐6, an increase in the number of synapse‐like protrusions and improved synaptic growth, a decrease in the spontaneous firing rate has been recorded.^[^ ^177^ ^]^ Therefore, IL‐6 may be the culprit that prevents neuronal growth and leads to neuronal defects.

Changes in synapses disrupt the balance between excitatory and inhibitory signaling. Therefore, the ASD model with MEA in vitro, Qt‐PCR, western blot, and other technologies to explore the effect of synaptic connections on the balance of excitation and inhibition of neural networks and the onset of ASD. In the above experiments, commercial MEAs have been used in most of the ASD models in vitro, and the number of wells and electrode sites in each MEA is small. It caused the ability to detect the release of neural networks is limited, and the data needs to be integrated and analyzed manually. Hence there is an urgent need for a fast, high‐throughput MEA solution that can automate comprehensive data analysis. In the future, hiPSC‐DNs can be cultured on novel MEAs, which are more beneficial for large‐scale long‐term detection and analysis. For the introduction of novel MEAs, please refer to Part 4.

### 3.3 Burst and Spike Analysis of Other hiPSC Models Recorded by MEA

#### 3.3.1 Number of Spikes in Pain Models

Long‐term taking or abuse of chronic painkillers may give rise to tolerance and side effects in the human body.^[^ ^179^, ^180^ ^]^ Therefore, there is an urgent need for emerging drugs and therapies that improve efficacy and reduce adverse reactions. Inducing hiPSC‐DNs to differentiate into sensory neurons and observing their phenotypic characteristics is considered to be an effective way to infer human pain perception.^[^ ^181^ ^]^ For example, when individuals in chronic pain, capsaicin dorsal root ganglion (DRG) sensory neurons showed excitability,^[^ ^182^ ^]^ and the excitability can be characterized by electrophysiological parameters such as MFR, NBR recorded by MEA. In addition, nociceptive stimuli are converted into nerve impulses by nociceptors. Therefore, peripheral neurons, as multimodal pain nociceptors, will respond to thermal, chemical, mechanical, and other stimuli to induce pain and produce neurophysiological responses.^[^ ^183^, ^184^, ^185^ ^]^

**Figure 7:**
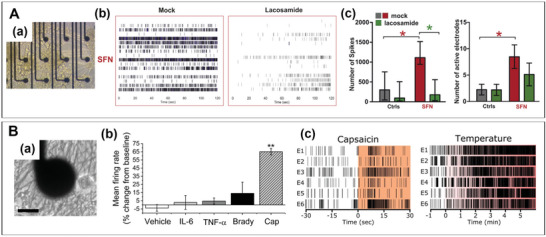
Microelectrode arrays (MEAs) recording results in pain models. A) Patient‐induced pluripotent stem cell (piPSC) derived nociceptors were recorded by MEA.^[^ ^186^ ^]^ a) Nociceptorson MEA chip; b) Raster plot examples of spontaneous activity of small fiber neuropathy (SFN) piPSC‐derived nociceptors in MEA with 500 × 10^−6^ m lacosamide (right side) over 120 s. c) Quantification of the number of spikes, active electrodes during 2 min recordings. B) Antinociceptive models recorded by MEA.^[^ ^192^ ^]^ a) Phase contrast image of dense cellular and axonal network on MEA substrate after 48 days in vitro. Scale bar represents 20 µm. b,c) Human‐induced pluripotent stem cell (hiPSC) sensory neuron firing rates were insensitive to human recombinant IL‐6, TNF‐α, and bradykinin (Brady), but were significantly increased by the addition of 100 × 10^−9^ m capsaicin. A) Reproduced with permission.^[^ ^186^ ^]^ Copyright 2019, Elsevier. B) Reproduced with permission.^[^ ^192^ ^]^ Copyright 2019, Elsevier.

Pain models can be established to explore the corresponding painkillers. For example, Barbara et al. acquired patient fibroblasts to reediting and differentiate into nociceptors to construct a small fiber neuropathy sensory perception model.^[^ ^186^ ^]^ In the model, the proportion of spontaneous activity of nociceptive fibers in neural activity was significantly increased. This spontaneous activity is thought to be a major cause of neuropathic pain.^[^ ^187^, ^188^ ^]^ Then, the lacosamide was tested in this model and reduce the spontaneous neural activity (**Figure** **7**Ab). It may due to lacosamide specifically interfere with peripheral sodium channels expressed by hiPSC‐derived sensory neurons, such as Nav1.7, Nav1.8, and Nav1.9.^[^ ^189^, ^190^ ^]^ The conclusions obtained from the piPSC‐derived nociceptors in vitro model were then applied to clinical treatment. Obviously, Spontaneous activity of piPSC‐derived nociceptors in MEA is significantly reduced by lacosamide application including the number of spikes in a burst, MFR, NBR, and BD (Figure 7A). Surprisingly, the receptor activity in the patient's microneurography was reduced, and the patient's pain was significantly reduced.^[^ ^186^ ^]^ In addition, some drugs for chemotherapy‐induced peripheral neuropathy, such as cisplatin, sunitinib, colchicine, and CC‐90003181, cause neuronal toxicity and damage the DRG may also cause pain. Meanwhile, the electrophysiological parameters of MFR and wMFR in hiPSC‐DNs were significantly increased. And Wainger et al. found that both capsaicin and mustard oil application evoked robust action potential firing from the induced neurons by culturing the hiPSC‐DNs on MEAs.^[^ ^191^ ^]^ Since capsaicin is often associated with nociceptive sensation, capsaicin and mustard oil may be potential nociceptive agents to study in the next nociceptive model. Subsequently, Black et al. recorded the electrophysiological signal of hiPSC‐derived sensory neurons by MEA. Compared to the control group with the other compounds, such as IL‐6, TNF‐ɑ, Brady, the MFR of the hiPSC‐derived sensory neurons was significantly increased by the addition of 100 × 10^−9^ m capsaicin (Figure 7Bb), which indicated hiPSC‐derived sensory neurons were sensitive to capsaicin. In addition to capsaicin‐induced nociceptive electrophysiological signals that can be captured by MEA, electrical, chemical, and thermal have also been shown to act as inducers of nociceptive factors (Figure 7B).^[^ ^192^ ^]^

At the same time, many alkaloids are the source of analgesics, and alkaloids are rarely used in pain models in current models. Therefore, to study the mechanism and pharmacological effects of chronic pain more comprehensively, it is necessary to further optimize the culture conditions or differentiation scheme, and establish a model of hiPSC‐derived sensory neurons sensitive to inflammatory factors. In addition, the use of MEA to record hiPSC‐DN pain models is limited, and subsequent models can be expanded around inflammatory pain, cancer pain, and neuropathic pain (eg, trigeminal neuralgia).

#### 3.3.2 Neural Electrical Activities of hiPSC‐DNs Parkinson and ALS Models

In addition to the above‐mentioned neural disease, models of Parkinson's, motor neuron ALS, and other rare diseases may also be blame for endogenous gene mutation and exogenous compound influence. Therefore, hiPSC‐DN models are also helpful for targeted research on these diseases.

The hiPSC‐DNs Parkinsonian model established in vitro can be used to treat Parkinsonian symptoms caused by LRRK2/PRKN mutations. Lebedeva et al. cultured dopamine neurons in vitro and transplanted them into the striatum to improve motor function and alleviate Parkinson's symptoms.^[^ ^193^ ^]^ They obtained fibroblasts from the skin of three patients with inherited Parkinson's disease with mutations in the LRRK2 and PRKN genes to establish a model of dopaminergic neurons expressing tyrosine hydroxylase on MEA.^[^ ^193^ ^]^ After 6‐OHDA in vitro treatment, the single spikes and bursts of spikes in neural network activity were recorded by MEA in real time, and other neurophysiological status of the cultured piPSC‐DN with Parkinson's were monitored.^[^ ^193^ ^]^ The recorded data was compared with healthy hiPSC‐DNs. After it was determined that the electrophysiological parameters of the two piPSC‐DNs were not be distinguishable from hiPSC‐DNs in healthy individuals. Then the piPSC‐derived dopamine neurons were transplanted into the striatum of patients. Subsequently, treated Parkinson's patients who received the treatment showed marked improvement in motor function.^[^ ^193^ ^]^ It could pave the way for hiPSC‐DNs treatment in vitro for Parkinson's disease.

Since the neurophysiology features of ALS patients are generally characterized by the action potentials of motor neurons, and the action potential of motor neurons can be represented by hiPSC‐derived motor neurons in vitro. Thus, the model of ALS established in vitro on MEA is more widely used for therapeutic screening than other motor nerve diseases.^[^ ^194^ ^]^ The C9ORF72RE,^[^ ^195^ ^]^ SOD1‐G93A,^[^ ^196^ ^]^ and SOD1A4V/+^[^ ^197^ ^]^ are all related genes that cause ALS which are summarized in **Table** **2**. Among them, Perkins et al. cultured piPSC‐derived ALS with C9ORF72RE mutation to study the influence of C9ORF72RE mutant gene on ALS.^[^ ^195^ ^]^ The C9ORF72RE mutation recorded by MEA resulted in increased network burst activity and decreased BD, and the motor neurons derived from patients showed significant hyperexcitability in both total firing rate and MFR.^[^ ^195^ ^]^ In addition, SOD1 mutation is also a common cause of ALS. The SOD1 gene encodes an antioxidant enzyme that binds copper ions and zinc ions to destroy superoxide free radicals (O^2−^) in the body and play an antioxidant role.^[^ ^198^ ^]^ Kim et al. used targeted gene editing technology to edit hiPSCs to differentiate into motor neurons containing SOD1‐G93A missense mutations. Structurally, the mutant gene resulted in enlarged, shorter synapses and fewer numbers of branch points. In terms of electrophysiological performance, it was observed that the number of neuron spikes decreased, the neural network burst disappeared, and the spike duration was prolonged.^[^ ^196^ ^]^ In conclusion, the use of patient‐derived ALS models can be used to study the effect of specific genes on ALS.

**Table 2:** Disease models with microelectrode arrays (MEAs).

Model	Source of Cells	Causative factor	Reported MEA metrics	Combined Method	Ref.
Epilepsy	piPSC‐DN and piPSC‐derived neural precursor cells	ACTL6B mutation	MBR MFR	Protein structure prediction RT‐PCR Western blot ACTL6B Antibody Validation Immunocytochemistry Protein Stability Assay MEA	[156]
	piPSC‐derived cortical neurons	m.3243A>G mutation	MFR MNBR PRA PTA	Droplet Digital PCR Patch‑Clamp Whole‑Cell Recordings Mitochondrial Morphology MEA	[155]
	piPSC‐derived GABAergic neurons	STXBP1‐E mutation	BD MFR NB NS	Quantitative RT‐qPCR immunocytochemistry Western Blot MEA	[157]
	hiPSC‐derived cortical neurons	‐	Duration and Nov. of spikes in a SBF MFR SBF	Immunocytochemistry MEA	[239]
ASD	piPSC‐DN	ACTL6B mutation	MBR MFR	Behavioral Assessment of Mouse ACTL6B Mutants Transcriptional and Proteome Profiling MEA	[240]
	piPSC‐derived GABAergic neurons	CNTN5^−/+^or EHMT2^−/+^ mutation	MFR NNB wMFR	Gene Editing Patch‑Clamp Whole‑Cell Recordings Western Blot Mycoplasma Testing MEA	[170]
	piPSC‐DN	TSC2 mutation	MFR Nov. of SB SB firing rate SB length %spikes firing outside of a SB	RNA Extraction Quantitative (q)PCR MEA	[169]
	piPSC‐3D cortical spheroids	Hyaluronan	wMFR	MEA	[166]
Parkinson	piPSC‐dopaminergic neurons	LRRK2/PRKN	NS	MEA	[193]
ALS	piPSC‐derived cortical neurons	C9ORF72^RE^	Inter‐burst length MBD MFR	Patch‑Clamp Whole‑Cell Recordings Immunocytochemistry Pharmacology and Transcriptomic Profiling. MEA	[195]
	piPSC‐DN	SOD1‐G93A	ANBD ANS MFR NNB MNBR NS	Patch‑Clamp Whole‑Cell Recordings Immunocytochemistry Gene Editing Western Blotting Microscopy and Image Acquisition MEA	[196]
	piPSC‐derived Motor Neurons	SOD1^A4V/+^	ANS MFR NAN	Patch‑Clamp Whole‑Cell Recordings MEA	[197]

Average network burst duration‐AND, Average number of spikes‐ANS, Burst duration‐BD, Mean network burst duration‐ NBD, Mean burst duration‐MBD, Mean burst rate‐MBR, Mean firing rate‐MFR, Mean network burst rate‐MNBR, Number of active neurons‐NAN, Number of bursts‐NB, Number of network bursts‐NNB, Number of spikes‐NS, Percentage of random activity‐PRA, Percentage of total activity‐PTA, Number of synchronized burst firing‐SBF, Weighted mean firing rate‐wMFR.

**Table 3:** Detection drugs for rodent primary neurons or human‐induced pluripotent stem cell derived neurons (hiPSC‐DNs).

Compound	The main mode of action	Detection object	Ref.
Picrotoxin	GABA_A_ receptor antagonist	hiPSC‐DN	[143]
Bicuculline		Rodent/hiPSC‐DN	[106, 144, 241–246]/[239, 246–250]
GABA	GABA_A_ receptor agonist	Rodent/ hiPSC‐DN	[244, 251–254]/[248, 255]
tetrodotoxin	Na^+^ channel antagonist	Rodent/ hiPSC‐DN	[144, 242, 256, 257]/[248, 250, 258–260]
Phenytoin		Rodent/ hiPSC‐DN	[142, 252]/[239]
4‐Amino‐pyridine	K^+^ channel antagonist	Rodent/ hiPSC‐DN	[142, 144, 145, 261]/[148, 249, 262]
Memantine ^+^NBQX	NMDAR and AMPAR antagonist	Rodent/ hiPSC‐DN	[263, 264]/[259, 260]
Carbenoxolone	Gap junction inhibitor NMDAR antagonist	Rodent/ hiPSC‐DN	[265, 266]/[247]

hiPSC‐DNs on the MEA can be used to establish acute neurodegenerative disease models, such as cerebral ischemic stroke models;^[^ ^199^ ^]^ chronic neurodegenerative disease models, such as Parkinson's disease models; motor neuron disease models, such as ALS, spinal muscular atrophy,^[^ ^200^ ^]^ spinal and bulbar muscular atrophy, and hereditary spastic paraplegia hereditary spastic paraplegia.^[^ ^201^, ^202^, ^203^ ^]^ The establishment of various disease models is beneficial to study the pathogenic mechanism of the disease, including patient endogenous gene mutations, and exogenous compound changes. Moreover, piPSC‐DN neural disease models can be established according to the patient's pathogenesis to propose reasonable treatment methods and drug. The piPSC‐DN disease model is cultured on the MEA, which enables high‐throughput, specific electrophysiological activity detection of neurons or neural networks. For some rare diseases, such as Kleefstra syndrome, Dravet syndrome, and Smith Lemli Opitz syndrome, the hiPSC models are closer to simulating brain development and pathology than animal experiments and primary animal cell culture. More accurately predict patient response. The research on the pathogenesis of psychological diseases such as depression and emotional eating disorders is also one of the directions that hiPSCs can expand in the MEA in the future, which will bring hope to more neural disease treatments.

#### 3.3.3 Field Potential and Burst of hiPSC‐DNs Exposed to Neurotoxic Substances

hiPSC‐DNs on the MEA in vitro can be used to determine the use and effect of novel neurotoxic substances, to clarify the direction of future research and development of such substances.^[^ ^204^ ^]^ For example, Hondebrink et al. established a co‐culture model of hiPSC‐DNs with astrocytes and hiPSC‐derived dopamine neurons, 100 × 10^−6^ m methoxetamine could inhibit neuronal activity to 14.2% and 5%, indicating that methoxetamine can strongly inhibit neuronal activity.^[^ ^205^ ^]^ Sonlicromanol has been shown to improve neuronal network activity and gene expression upon early treatment. Tukker et al. established a co‐culture model of hiPSC‐glutamatergic neurons and astrocytes to detect the effects of endosulfan and neurotoxic methylmercury on ​​different parameters of neuronal activity. It was found that picrotoxin reduced MBR, while thiophene and methylmercury had no significant effect on MBR. From this, it is reasonable to judge the appropriate concentration of the drug to be used.^[^ ^206^ ^]^ It is helpful to improve the screening efficiency, reduce the complicated experimental steps in animal experiments, and improve the efficiency of the early‐stage development of drug screening.

There are a wide variety of neurotoxic substances for neural diseases and their classification is complex. However, the principles of these substances acting on the nerves or nervous system are mostly the same, therefore it can be classified by the principle of action and effect. The MEA‐based electrophysiological model of Tixier et al. evaluated drug properties based on the morphology, duration, and amplitude of repolarizing and depolarizing waves in field potentials. Neurotoxic substances were classified by blockage of cellular ion channels.^[^ ^207^ ^]^ Currently, picrotoxin, bicuculline, tetrodotoxin, and other neurotoxic substances have been tested with hiPSC‐DNs, as shown in **Table** 3. In addition, pentylenetetrazol, amoxapine, and endosulfan can also be used for hiPSC‐DN model testing as a supplement to animal experiments.

## 4 Conclusions and Outlook

### Multifunction Coupling in MEA

**Figure 8:**
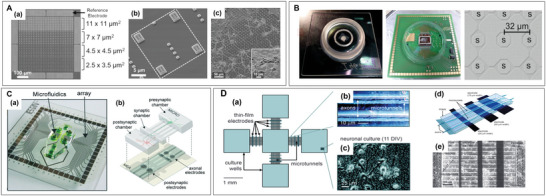
Microelectrode arrays (MEAs) combined with CMOS and microfluidic could be used in human‐induced pluripotent stem cell derived neurons (hiPSC‐DNs) in the future. A) The morphology, surface, and circuit of CMOS‐MEA.^[^ ^237^ ^]^ a) SEM image of one active area featuring the four different electrode sizes and the eight reference electrodes. b) SEM images of a single‐pixel containing electrodes of sizes 2.5 × 3.5 µm^2^. c) SEM images of a 3 DIV primary hippocampal neuron culture on a single active area and a zoomed region (inset) of the CMOS chip. B) Pictures of the 256‐CMOS‐MEA, which can record human cerebrospinal fluid.^[^ ^238^ ^]^ C) Microfluidic‐MEA for the study of activity‐dependent intracellular dynamics in neuronal networks.^[^ ^217^ ^]^ a) The structure of the microfluidic‐MEA platform. b) Scheme showing the microfluidic (micro) and microelectrode (MEA) components of the platform. D) A novel MEA platform with PDMS microchannels enables the detection of action potential propagation from isolated axons in culture.^[^ ^215^ ^]^ a) Schematic of the design configuration. b) Phase‐contrast image of axonal growth inside micro tunnels. c) Healthy neuronal growth inside culture well. A) Reproduced under terms of the CC‐BY license.^[^ ^237^ ^]^ Copyright 2019, The Authors. Published by Frontiers Media S.A. B) Reproduced under terms of the CC‐BY license.^[^ ^238^ ^]^ Copyright 2020, The Authors. Published by Frontiers Media S.A. C) Reproduced with permission.^[^ ^217^ ^]^ Copyright 2018, Royal Society of Chemistry. D) Reproduced with permission.^[^ ^215^ ^]^ Copyright 2009, Royal Society of Chemistry.

Currently, MEA is advancing toward multifunctional and integrated devices. The combination of MEA with CMOS technology serves as a prominent example of this development. (**Figure** **8**A,B).^[^ ^208^, ^209^, ^210^, ^211^ ^]^ The CMOS‐MEA, higher density arrays allow for more accurate spike sorting and determination of single neuronal units.^[^ ^212^ ^]^ Meanwhile, noise reduction as well as the stabilization and amplification of the signal are performed to reduce the workload of postsignal processing. For example, CMOS‐high density‐MEA has been shown to detect retigabine on motor neuron activity, providing new research options for the discovery of therapeutic drugs for Parkinson's and ALS.^[^ ^211^ ^]^ Further high‐density CMOS‐MEA enhances the accuracy of neural signals detection, allowing for the assessment of compound effects at the cellular level. Additionally, when combined with microfluidic technology, microchannels within the microfluidic system enable precise drug delivery to neurons, enhancing the selectivity and spatial resolution of delivered compounds (Figure 8C,D).^[^ ^213^, ^214^, ^215^, ^216^, ^217^ ^]^ The microchannels also constrain the growth direction of neurons, allowing experimentalists to engineer the topology of neuronal circuits in vitro, as opposed to random circuits that arise using traditional 2D cultures. Therefore, MEA is gradually integrating microfluidic, CMOS, and other technologies, so that the detection platform develops in the direction of miniaturization and multifunction.

### 3D Organoid Detecting by MEA

Given that neurons in the brain possess a spatial structure, there are ongoing international efforts to explore 3D organoid cultures.^[^ ^218^ ^]^ 3D culture allows for modeling regional connectivity between different brain regions and organs. The first generation of such on‐chip body models already exist.^[^ ^219^ ^]^ The Sasai laboratory made groundbreaking discoveries regarding the self‐organizing properties of brain organoids, instilling confidence in establishing in vitro 3D organoid cultures.^[^ ^220^, ^221^ ^]^ However, the physiological processes that brain organoids can accurately mimic remain unclear. Fortunately, Giorgia et al. found that optogenetic stimulation can be employed to control neuronal activity in organoids, enabling the simulation of macroscopic sensory activities in 2017.^[^ ^222^ ^]^ Additionally, the simulation of detailed neural system activities, including long‐range projections both within and beyond organoids, growth cone rotation, and decussation, was achieved in 2019.^[^ ^223^ ^]^ The emergence of the 2020 slice‐based neocortex organoid system mitigated cell death caused by internal hypoxia and insufficient surface diffusion, further advancing brain organoid cultivation.^[^ ^63^ ^]^ At this stage, brain organoid cultivation has become relatively refined, but there is still a need for further exploration in detection methods. In 2020, the developmental trajectory of brain organoids was shown to be recordable by MEA, capturing electrophysiological activity corresponding to cell morphologies,^[^ ^224^ ^]^ and applied in disease model analyses.^[^ ^34^ ^]^ More recently, Neuropixels probes and HD CMOS‐based MEAs were utilized by Sharf et al. to map neuronal circuits in brain organoids, further enhancing the analysis of brain organoids.^[^ ^34^ ^]^ This system was used to examine the effects of neuropsychiatric compounds on human‐derived networks.^[^ ^34^ ^]^

**Figure 9:**
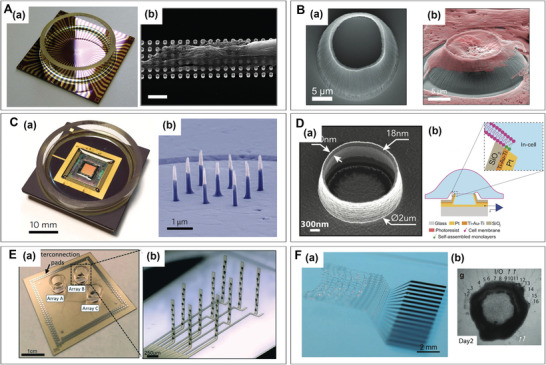
3D microelectrode arrays (MEAs) could be used in human‐induced pluripotent stem cell derived neurons (hiPSC‐DNs) in the future. A) 3D mushroom‐shaped electrode. a) 64 electrode MEAs exhibiting an 8 × 8 electrode grid;^[226]^ b) SEM images of a guided HL‐1 cell on nanopillars with an interspace of 2 µm (scale bar 5 µm).^[^ ^225^ ^]^ B) 3D Microstructured carbon nanotube MEA.^[^ ^227^ ^]^ a) SEM images of a 20 µm microwell. b) Colored SEM image of a primary cardiomyocyte within the cavity of a CNT well. C) 3D‐CMOS‐MEA.^[^ ^229^ ^]^ a) The device is wire‐bonded to a chip carrier: a glass ring and a polydimethylsiloxane protective layer create a microfluidic well. b) False‐colored scanning electron microscope image of nine vertical nanoelectrodes fabricated per pad. D) Nanopatterned volcano‐shaped microelectrode arrays.^[^ ^38^ ^]^ a) SEM image of the microelectrode. b) Schematic drawing showing the overall structure of the nanovolcano and the interface to a cell. The dashed lines represent the junctional cell membrane at the cell–electrode interface. E) Bending 3D‐MEA.^[^ ^228^ ^]^ a) A completed device. The overall length and width, as well as pad locations, are identical to a commercial multichannel systems brand device, allowing for seamless integration into existing electrophysiology electronics. b) Light micrograph of a single 3D‐MEA postactuation. The hinge regions are plastically deformed and allow the probes to stand upright without additional support. F) 3D Stretchable Mesh MEA.^[^ ^100^ ^]^ a) Optical photograph of stretchable mesh nanoelectronics released from the substrate and floating in the saline solution. b) Black numbers and arrows indicate the input/output (I/O) stretchable connectors for the 16‐channel electrode array. The white arrows highlight the stretchable anchors used to keep the stretchable mesh nanoelectronics unfolded on the substrate, which was released after seeding with cells. A) Reproduced with permission.^[^ ^226^ ^]^ Copyright 2017, IOP Publishing Ltd. B) Reproduced with permission.^[^ ^227^ ^]^ Copyright 2017, Wiley‐VCH. C) Reproduced with permission.^[^ ^229^ ^]^ Copyright 2017, Springer Nature. D) Reproduced under terms of the CC‐BY license.^[^ ^38^ ^]^ Copyright 2019, The Authors. Published by American Chemical Society. E) Reproduced under terms of the CC‐BY license.^[^ ^228^ ^]^ Copyright 2020, The Authors. Published by Royal Society of Chemistry. F) Reproduced with permission.^[^ ^100^ ^]^ Copyright 2022, Wiley‐VCH.

At present, the technical means to detect 3D culture are limited, and MEA is currently developing in the direction of 3D. For example, 3D detection of electrodes can be realized by modifying 3D nanomaterials and bending electrodes to form spatial structures. Mushroom‐shaped electrodes can enhance the electrical coupling between neurons and electrodes (**Figure** **9**A);^[^ ^225^, ^226^ ^]^ membrane‐wrapped volcano‐shaped electrodes can directly record intracellular signals (Figure 9B),^[^ ^38^, ^227^ ^]^ and bent 3D electrodes form spatial structures that can cover 3D cultured brain organoids (Figure 9E).^[^ ^228^ ^]^ Upright nanowire electrodes can be used to record intracellular or extracellular signals (Figure 9C,D).^[^ ^229^, ^230^, ^231^, ^232^ ^]^ Au‐modified hollow nanotubes can directly transport drugs into cells, and simultaneously detect intracellular signals.^[^ ^213^, ^233^ ^]^ At present, few schemes are using 3D electrodes to capture in vitro organoid detection signals, and in the future, they can be used for drug screening and the establishment of in vitro neurological disease models (Figure 9F).^[^ ^100^, ^214^, ^234^, ^235^ ^]^ Therefore, the transformation from 2D to 3D structure is a method to change the MEA interface morphology to improve the MEA interface properties, which is helpful for the study of neuronal organoid models.

### Brain Organ‐on‐a‐Chip

The transition from 2D hiPSC culture to the establishment of 3D brain organoid models has led to the gradual development of in vitro human brain constructs in the field of bioengineering. Currently, the integration of human brain pericytes, astrocytes, and neurons has been achieved in organ‐on‐a‐chip platforms.^[^ ^236^ ^]^ Additionally, Maoz et al. fabricated an organ‐on‐a‐chip to model the human neurovascular unit in vitro.^[^ ^20^ ^]^ MEA, microfluidics, and CMOS technologies are enabling multifunctional coupling, with microfluidics facilitating spatial structure cultivation and physiochemical signal transmission, 3D microelectrode arrays achieving high‐resolution spatiotemporal electrophysiological signal transmission and recording, and CMOS‐MEA technology integrating high‐throughput signal detection and processing. Further connections between biology and digital technology are being established, enabling the interface of brain organoids with real‐world sensors and output devices, creating a biofeedback loop. Utilizing artificial intelligence algorithms for training, this advancement aims to ultimately achieve an in vitro lifelike simulation of brain activity.^[^ ^125^ ^]^

The detection of hiPSC‐DNs on MEAs has a human genetic background, which can be used for targeted disease research on patients, and the neural network of patients with neural diseases can be established in vitro in 3D, and then transplanted into the injured nervous system, therefore patients with rare diseases can benefit from targeted diagnosis and treatment. However, in vitro disease diagnosis and drug screening still have limitations. For example, 3D organoids can only know the local effects of drugs in the brain, while the global effects in the patient's brain or body are still to be solved. In the future, the establishment of 3D hiPSC‐DNs is closer to the human neural network. Compared with a single type of cell culture, the effect of the drug on the target and the influence of the peripheral neural circuit can be detected macroscopically, which can greatly facilitate the precise treatment of drugs. However, in the future, if inBMI technology can be used to establish models with physiological characteristics similar to those of the human brain, accurate diagnosis and treatment may be possible.

## Conflict of interest

The authors declare no conflict of interest.
